# A Ka-Band Integrated Six-Port Chip for Analog Complex Correlator

**DOI:** 10.3390/s22134877

**Published:** 2022-06-28

**Authors:** Wangdong He, Xi Chen, Jianhao Gong, Anyong Hu, Jungang Miao

**Affiliations:** School of Electronics and Information Engineering, Beihang University, Beijing 100191, China; hwd19930315@buaa.edu.cn (W.H.); chenxi0913@buaa.edu.cn (X.C.); gongjh@buaa.edu.cn (J.G.); jmiaobremen@buaa.edu.cn (J.M.)

**Keywords:** interferometric passive imaging, analog complex correlator, six-port technology, amplifier, phase shifter

## Abstract

Six-port technology has been widely used in microwave systems, such as interferometric passive imaging. In this paper, an integrated Ka-band (32–36 GHz) six-port chip based on the 0.15-μm GaAs technology is designed and fabricated to simplify the circuit structure and miniaturize the volume of the imaging system. The designed chip integrates two amplifiers, two phase shifters, and a six-port circuit as part of an analog complex correlator. In this integrated chip, the crosstalk between the two amplifiers cannot be ignored. This paper analyzes the influence of the isolation between two amplifiers on the correlation results to guide the six-port chip design. In addition, considering that the radiometer system receives a broadband noise signal, the phase shifter needs to ensure that the phase shift range of each frequency point is the same under the same control conditions. Therefore, the phase shifter is designed with a high-pass and low-pass structure. The measurement results show that the isolation between the two amplifiers is greater than 20 dB, and the measured phase shift range and phase shift range error of the designed chip are 220° and 10°, respectively, with the control voltage varying from 0 to 1.5 V, which meets the requirements of the system.

## 1. Introduction

The basic work related to six-port technology was carried out in the 1970s, primarily by Engen and Hoer [1,2,3,4]. Since that time, six-port technology has developed rapidly and has been widely used in many fields, such as reflectometer [5,6], radar detection [7,8,9,10], antenna measurement [11,12], and interferometric passive imaging [13].

In the application of interferometric passive imaging, the correlator is the key component. Both analog and digital approaches can be applied to correlators. The digital correlator has the advantages of high stability and flexible configuration [14,15]. The analog correlator has the benefits of large continuum bandwidth, high sensitivity, low costs, and low power consumption [16,17,18,19]. Furthermore, the analog correlator is followed by a relatively simple data acquisition and processing subsystem.

The analog correlator can be implemented using either direct multiplication technology [18,19,20,21] or add-and-square technology [22,23]. The direct multiplication analog correlator is normally carried out using non-linear devices such as mixers or analog multipliers [24]. Due to the limitation of multiplier chips, the correlator has the disadvantage of low bandwidth and operates in the low-frequency range. The add-and-square analog correlator can achieve wide bandwidth and high sensitivity at the expense of larger volume and complex structure [25,26,27], and it is normally based on six-port technology [28]. In addition, the add-and-square analog complex correlator can obtain the amplitude and phase information of the two input signals by measuring the correlation of received signals. In order to obtain the complete correlation circle, the phase difference between the two input signals needs to be swept from 0° to 360°.

A six-port network, which has two inputs and four outputs, is a passive microwave system composed of couplers and power dividers [29,30,31]. The six-port network can be realized by microstrip technology [32,33,34,35], but it leads to the six-port being larger and inhibits the miniaturization of the correlator. Therefore, in order to increase the integration of the system, a six-port network fabricated on the chip, which may integrate other circuits, such as a detector or amplifier, has been reported in recent years [36,37,38,39,40]. The chip integration improves the accuracy and reduces the size of the six-port circuit.

**Figure 1:**
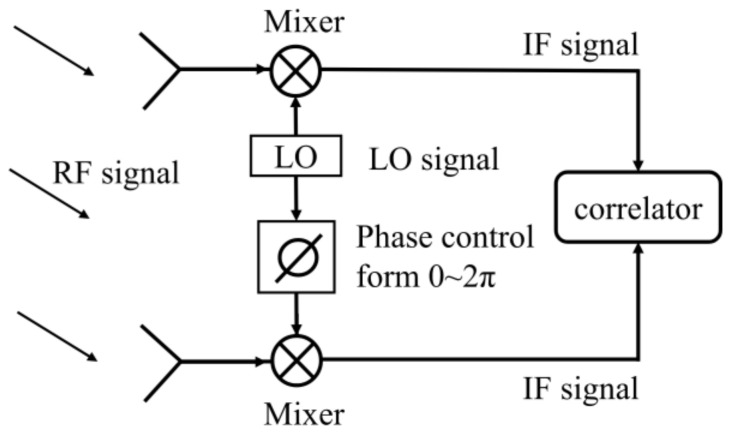
A simple block diagram of the interferometric imaging system.

In the application of interferometric millimeter-wave passive imaging, the higher input frequency means better spatial resolution, which is very important to the system [41,42]. In addition, the temperature sensitivity of a radiometer is inversely proportional to the square root of the system bandwidth [43]. The interferometric imaging system can easily obtain larger bandwidth at a higher frequency, thereby increasing the temperature sensitivity of the system. In the design of interferometric imaging systems, the receiver front-end works in the high-frequency range (such as the Ka-band), but its correlator works in a lower frequency band (such as C-band, 4–8 GHz) [15,44,45,46]. Therefore, a local oscillator (LO) chain is introduced into the system to down-convert the received radio frequency (RF) signal to obtain the intermediate frequency (IF) signal with a lower frequency. A simple block diagram of the interferometric imaging system is represented in Figure 1.

It is worth noting that the phase control module is designed in the LO chain, which is used to change the phase difference between two input RF signals from 0° to 360° to obtain a complete correlation circle in the correlator. However, this method adds an additional LO link and increases the complexity of the system. Especially for the passive millimeter-wave imaging system with a large number of receiver channels, it needs the same number of LO signals, and the LO chain is a burden to the system that cannot be ignored.

**Figure 2:**
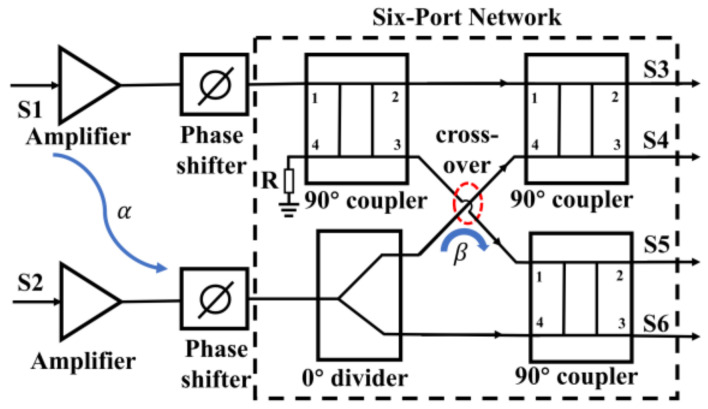
The block diagram of the proposed six-port chip integrated with two amplifiers, two phase shifters, and a six-port circuit.

In this article, a Ka-band integrated six-port chip is designed and fabricated based on 0.15-µm GaAs pHEMT technology. The designed chip integrates two amplifiers, two phase shifters, and a six-port circuit as part of an analog complex correlator, and it is used for interferometric passive millimeter-wave imaging applications [47]. The block diagram of the proposed integrated six-port chip is represented in Figure 2. In this structure, two amplifiers at two input ports are used to provide gain to the input RF signals, which can compensate for the signal loss caused by the phase shifter and six-port network. The phase shifter is a phase control module to change the phase of input RF signals, and the six-port network comprised of a power divider and 3-dB hybrid couplers is used to shift the signal phases and addition of the input signals. The theory of the complex cross-correlation circuits is described, and the influence of the isolation between two amplifiers on the correlation results is analyzed to guide the integrated six-port chip design. The phase shifter with low-pass and high-pass structure is simulated and designed, which has the characteristics of broadband phase-shifting compared with the phase shifter with only low-pass structure. Based on the designed integrated six-port chip, the measurement isolation between two amplifiers and phase shift range is performed and analyzed.

The remainder of this article is structured as follows. In Section 2, we describe the theory of the complex cross-correlation circuits, after which the influence of the isolation between two amplifiers is given to guide the six-port circuit design. Section 3 presents the design of the integrated six-port chip, including the design of each single function circuit and the simulation of the whole integrated circuit. In Section 4, the measurement results employing the integrated six-port chip are presented. The conclusion is provided in Section 5.

## 2. Analysis of the Analog Complex Correlator Based on the Integrated Six-Port Chip

**Figure 3:**
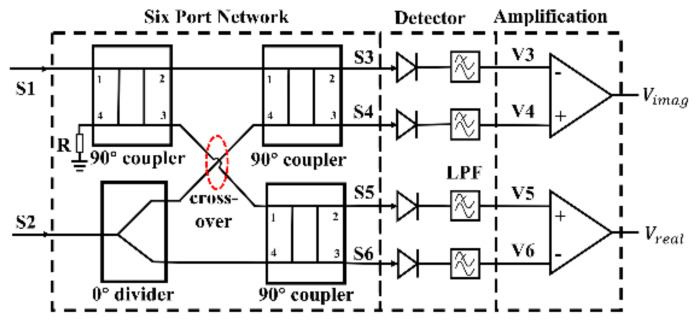
Topology of the complex cross-correlator circuit based on six-port technology.

In the interferometric passive imaging system, an ideal analog complex cross-correlator is usually used to measure the phase difference between two input signals. The six-port cross-correlator can obtain the phase difference by first adding and then squaring the two input signals. In our team's previous work [28,45], the analog complex cross-correlator based on the six-port technology, shown in Figure 3, was applied in a passive imaging system for security applications. It can be seen that the six-port network consists of a power divider and three couplers.

### 2.1. Theory of the Analog Complex Correlator

This section introduces the theory of the analog complex correlation circuits in an ideal case. Assuming the inputs are two single-frequency signals $S_{1}\left(t\right)$ and $S_{2}\left(t\right)$, which can be presented as:

(1) $$\begin{array}{l} S_{1}\left(t\right)=\cos\left(\omega_{0}t+\theta_{1}\right)\\ S_{2}\left(t\right)=\cos\left(\omega_{0}t+\theta_{2}\right) \end{array}$$

Then, the four output signals of the six-port network can be described as:

(2) $$\begin{array}{l} S_{3}=S_{1}+{\hat{S}}_{2}=\cos\left(\omega_{0}t+\theta_{1}\right)+\sin\left(\omega_{0}t+\theta_{2}\right)\\ S_{4}={\hat{S}}_{1}+S_{2}=\sin\left(\omega_{0}t+\theta_{1}\right)+\cos\left(\omega_{0}t+\theta_{2}\right)\\ S_{5}={\hat{S}}_{1}+{\hat{S}}_{2}=\sin\left(\omega_{0}t+\theta_{1}\right)+\sin\left(\omega_{0}t+\theta_{2}\right)\\ S_{6}=-S_{1}+S_{2}=-\cos\left(\omega_{0}t+\theta_{1}\right)+\cos\left(\omega_{0}t+\theta_{2}\right) \end{array}$$

where ${\hat{S}}_{1}$ is Hilbert transform of $S_{1}$, which is used to represent the phase of signal shifted by 90° in the six-port signal distribution network.

The following detector circuits with the square-law characteristic and low pass filter (LPF) transform input signals ($S_{3}$–$S_{6}$) to useful output dc voltages ($V_{3}$–$V_{6}$). After the differential amplification circuits, the real and imaginary parts of the cross-correlation function can be obtained [28] as:

(3) $$\begin{array}{l} V_{real}=GV_{2nd}\cos\left(\theta_{1}-\theta_{2}\right)\\ V_{imag}=GV_{2nd}\sin\left(\theta_{1}-\theta_{2}\right) \end{array}$$

where *G* is the gain of the differential amplification circuit, and$\;V_{2nd}$ is the dc voltage resulting from the second-order transconductance of the square-law detector. In order to characterize the performance of the correlator, a correlation circle has been formed through the real and imaginary parts of the correlation results. In addition, the broadband characteristic of the correlator can be evaluated by using the correlation circles.

### 2.2. Influence of Isolation between Two Amplifiers on Correlation Results

In this article, the fabricated integrated six-port chip contains amplification, phase-shifting, and signal distribution circuits, as shown in Figure 2. Since these circuits are implemented on a chip with a small size, the effect of signal coupling on the correlation results has to be considered. In this section, the isolation between two amplifiers at the input port is mainly considered. Moreover, we can analyze the influence of the isolation between two amplifiers on the correlation results under the condition that other components of the correlator are ideal.

Assuming that the voltage coupling coefficient between two amplifiers is *α*, and it varies from 0 to 1. The two inputs are still single-frequency signals $S_{1}\left(t\right)$ and $S_{2}\left(t\right)$, then two input signals of six ports can be described as:

(4) $$\begin{array}{l} {S^{\prime }}_{1}=\sqrt{1-\alpha^{2}}S_{1}+\alpha S_{2}\\ {S^{\prime }}_{2}=\alpha S_{1}+\sqrt{1-\alpha^{2}}S_{2} \end{array}$$

In this condition, the four output signals of the six-port network can be described as:

(5) $$\begin{array}{l} {S^{\prime }}_{3}=\sqrt{1-\alpha^{2}}\left(S_{1}+{\hat{S}}_{2}\right)+\alpha \left({\hat{S}}_{1}+S_{2}\right)\\ {S^{\prime }}_{4}=\sqrt{1-\alpha^{2}}\left({\hat{S}}_{1}+S_{2}\right)+\alpha \left(S_{1}+{\hat{S}}_{2}\right)\\ {S^{\prime }}_{5}=\sqrt{1-\alpha^{2}}\left({\hat{S}}_{1}+{\hat{S}}_{2}\right)+\alpha \left({\hat{S}}_{1}+{\hat{S}}_{2}\right)\\ {S^{\prime }}_{6}=\sqrt{1-\alpha^{2}}\left(-S_{1}+S_{2}\right)+\alpha \left(S_{1}-S_{2}\right) \end{array}$$

After the zero-biased square-law detector and LPF, the useful output dc voltage can be expressed as:

(6) $$\begin{array}{l} {V^{\prime }}_{3}=\frac{V_{2nd}}{2}\left[1-\left(1-2\alpha^{2}\right)\sin\left(\theta_{1}-\theta_{2}\right)+2\alpha \sqrt{1-\alpha^{2}}\cos\left(\theta_{1}-\theta_{2}\right)\right]\\ {V^{\prime }}_{4}=\frac{V_{2nd}}{2}\left[1+\left(1-2\alpha^{2}\right)\sin\left(\theta_{1}-\theta_{2}\right)+2\alpha \sqrt{1-\alpha^{2}}\cos\left(\theta_{1}-\theta_{2}\right)\right]\\ {V^{\prime }}_{5}=\frac{V_{2nd}}{2}\left[\left(1+2\alpha \sqrt{1-\alpha^{2}}\right)\times \left(1+\cos\left(\theta_{1}-\theta_{2}\right)\right)\right]\\ {V^{\prime }}_{6}=\frac{V_{2nd}}{2}\left[\left(1-2\alpha \sqrt{1-\alpha^{2}}\right)\times \left(1-\cos\left(\theta_{1}-\theta_{2}\right)\right)\right] \end{array}$$

Then, the real and imaginary parts of the cross-correlation function can be obtained as

(7) $$\begin{array}{l} V_{real}=GV_{2nd}\left[2\alpha \sqrt{1-\alpha^{2}}+\cos\left(\theta_{1}-\theta_{2}\right)\right]\\ V_{imag}=GV_{2nd}\left(1-2\alpha^{2}\right)\sin\left(\theta_{1}-\theta_{2}\right) \end{array}$$

Compared to the ideal case, it can be seen that the isolation between two input amplifiers has an influence on the amplitude of the imaginary part of the cross-correlation function and adds a dc offset error to the real part of the cross-correlation function. In addition, the relationship between isolation (*ISO*) and voltage coupling coefficient α can be expressed as:

(8) $$ISO=-20\log\left(\alpha \right)$$

**Figure 4:**
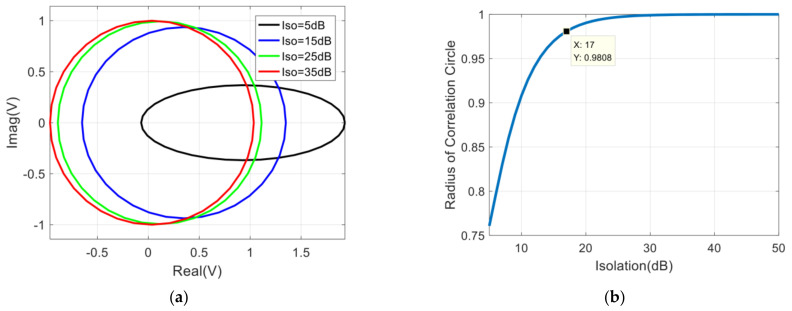
Influence of isolation between two amplifiers on correlation results: (**a**) The variation of the correlation circle with the isolation; (**b**) The variation of the radius of the correlation circle with the isolation.

According to Equations (7) and (8), the variation of the correlation circle with the isolation can be obtained, and then the radius of the correlation circle can be fitted according to the least square method, and the result is shown in Figure 4.

Since the dc offset error does not vary with the phase difference of the input signal ($\Delta \theta =\theta_{1}-\theta_{2}$), it can be calibrated by using the measurement results of the correlator by changing Δθ in the range of 2π at equal intervals [46]. Generally speaking, the correlation coefficient of the analog correlator is required to be greater than 0.9 [20,48]. In order to estimate the demand of the isolation between two input signals for the correlator quickly, we can specify that the variation range of the correlator circle radius is less than 2%. In Figure 4b, it can be seen that the requirement of the isolation between two input signals is larger than 17 dB.

### 2.3. Influence of Phase Shift Range Error on Correlation Results

As represented in Figure 1, the phase control module is designed in the LO link. Therefore, the phase of the broadband IF signal can be changed by controlling the phase of the LO signal with a single frequency, and the requirements of this system for phase shift are relatively simple. In order to reduce the burden of LO link, a method of directly controlling the phase of RF signals is proposed in this paper, and the proposed phase control module needs to deal with the wideband noise signal. The designed phase shifter needs to ensure that the phase shift range (PSR) of each frequency point is the same under the same control conditions. However, this requirement is difficult to achieve, so this section analyzes the impact of phase shift range error (PSRE) on correlation results.

In the case of broadband range, PSR varies at different frequencies under the same control conditions, and it can be expressed as $\Phi \left(f\right)$, which is a function of frequency. Therefore, we can define the phase shift range error (PSRE) as:

(9) $$\Delta \Phi =\Phi_{max}\left(f_{1}\right)-\Phi_{min}\left(f_{2}\right)$$

where ΔΦ is the PSR of the phase shifter,$\Phi_{max}$ and $\Phi_{min}$ are the maximum and minimum phase shift range within the operating frequency band, respectively. It is worth noting that the phase of the RF signal needs to be shifted by 360° to get a complete correlation circle. Since there are two phase shifters on two input channels of the designed chip, the PSR of the designed phase shifter only needs to be greater than 180°.

In order to evaluate the influence of PSRE on the correlation results, it is necessary to analyze the broadband noise characteristics of the analog complex correlator based on six-port technology. The two wideband white noise signals input to the complex correlator can be equivalent to the superposition of multipoint frequency signals. Since the power spectral density of white noise is constant, for the convenience of analysis, we set the amplitude of all frequency points to 1 and the initial phase of two signals $\theta_{1}^{i}$ and $\theta_{2}^{i}$ both satisfy the uniform distribution of 0~2π. Then the two inputs signals can be expressed as

(10) $$\begin{array}{l} {\tilde{S}}_{1}\left(t\right)=\sum_{i}\cos\left(\omega_{i}t+\theta_{1}^{i}\right)\\ {\tilde{S}}_{2}\left(t\right)=\sum_{i}\cos\left(\omega_{i}t+\theta_{2}^{i}\right) \end{array}$$

According to Equations (1)–(3), the correlation results for the ideal case can be expressed as:

(11) $$\begin{array}{l} V_{real}=\sum_{i}GV_{2nd}\cos\left(\theta_{1}^{i}-\theta_{2}^{i}\right)\\ V_{imag}=\sum_{i}GV_{2nd}\sin\left(\theta_{1}^{i}-\theta_{2}^{i}\right) \end{array}$$

It is assumed that the phase difference of the two broadband noise signals varies with frequency as a constant; that is, the cross-correlation coefficient of the two broadband noise signals is 1. Equation (11) can be simplified as:

(12) $$\begin{array}{l} V_{real}=\sum_{i}GV_{2nd}\cos\left(\theta_{1}-\theta_{2}\right)\\ V_{imag}=\sum_{i}GV_{2nd}\sin\left(\theta_{1}-\theta_{2}\right) \end{array}$$

Due to the PSRE of the phase shifter, a phase shift error ($\varphi^{i}$) will be introduced into the correlation results. Then the correlation results can be expressed as:

(13) $$\begin{array}{l} V_{real}=\sum_{i}GV_{2nd}\cos\left(\theta_{1}-\theta_{2}+\varphi^{i}\right)\\ V_{imag}=\sum_{i}GV_{2nd}\sin\left(\theta_{1}-\theta_{2}+\varphi^{i}\right) \end{array}$$

**Figure 5:**
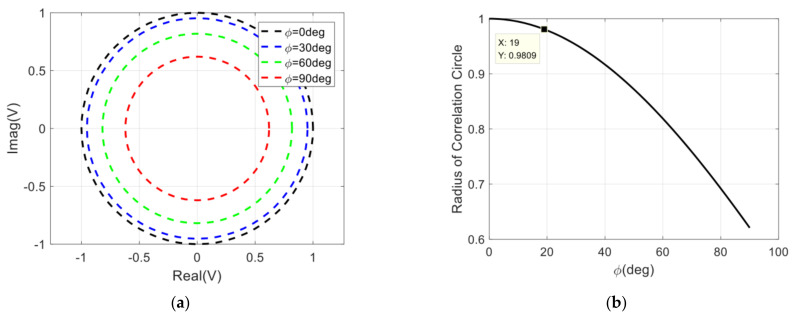
Influence of PSRE on correlation results: (**a**) The variation of the correlation circle with the PSRE; (**b**) The variation of the radius of the correlation circle with the PSRE.

The phase shift range of the designed phase shifter changes linearly with the frequency, which will be described in detail in the following section. Then we can assume that the value range of $\varphi^{i}$ is −ΔΦ/2~ΔΦ/2 under the condition of taking the center frequency as the reference. Therefore, the variation of the correlation circle and its radius with the PSRE can be obtained in Figure 5. In the same way, the requirement of the PSRE for the correlation system is ΔΦ≤19°.

### 2.4. Influence of Isolation in Cross-Over Structure on Correlation Results

As shown in Figure 2, a cross-over structure is used in the six-port network to keep the four outputs on the same side. Therefore it is necessary to evaluate the influence of isolation in the cross-over structure. Assuming that the voltage coupling coefficient in cross-over structure is β, and it varies from 0 to 1. The two inputs are still single-frequency signals $S_{1}\left(t\right)$ and $S_{2}\left(t\right)$, then the four output signals of the six-port network can be described as:

(14) $$\begin{array}{c} S_{3}=\left(1-\beta \right)S_{1}+\sqrt{1-\beta^{2}}{\hat{S}}_{2}\\ S_{4}=\left(1+\beta \right){\hat{S}}_{1}+\sqrt{1-\beta^{2}}S_{2}\\ S_{5}=\sqrt{1-\beta^{2}}{\hat{S}}_{1}+{\hat{S}}_{2}+\beta S_{2}\\ S_{6}=-\sqrt{1-\beta^{2}}S_{1}+S_{2}+\beta {\hat{S}}_{2} \end{array}$$

Refer to the derivation process in Section 2.2, after the detector and LPF, the real and imaginary parts of the cross-correlation function can be obtained as:

(15) $$\begin{array}{l} V_{real}=GV_{2nd}\sqrt{1-\beta^{2}}\cos\left(\theta_{1}-\theta_{2}\right)\\ V_{imag}=GV_{2nd}\left[\beta +\sqrt{1-\beta^{2}}\sin\left(\theta_{1}-\theta_{2}\right)\right] \end{array}$$

**Figure 6:**
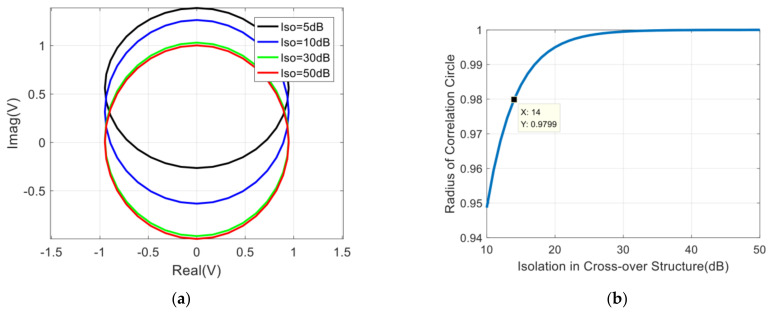
Influence of isolation in cross-over on correlation results: (**a**) The variation of the correlation circle with the isolation; (**b**) The variation of the radius of the correlation circle with the isolation.

Compared to the ideal case, it can be seen that the isolation in the cross-over structure has an influence on the amplitude of the real and imaginary part of the cross-correlation function and adds an additional dc offset error to the imaginary part. Then the variation of the correlation circle and its radius with the isolation in the cross-over structure can be obtained in Figure 6. In the same way, the requirement of the isolation in the cross-over structure for the correlation system is larger than 14 dB.

## 3. Design of the Integrated Six-Port Chip

As shown in Figure 2, the Ka-band (32–36 GHz) integrated six-port chip, which contains two amplifiers, two phase shifters, and a six-port network is analyzed and designed in this section. In order to reduce the difficulty of design and simulation, we design each single-function circuit first and then simulate the whole circuit. The WIN PL1512 GaAs pHEMT process, which has a nominal cutoff frequency of 95 GHz and features low noise performance, is chosen for the implementation of the circuit. In addition, due to the restriction of processing reticle rules, the size of the chip proposed in this paper is limited to 5 mm × 2 mm in order to be processed with other chips together.

### 3.1. Design of the Amplifier

**Figure 7:**
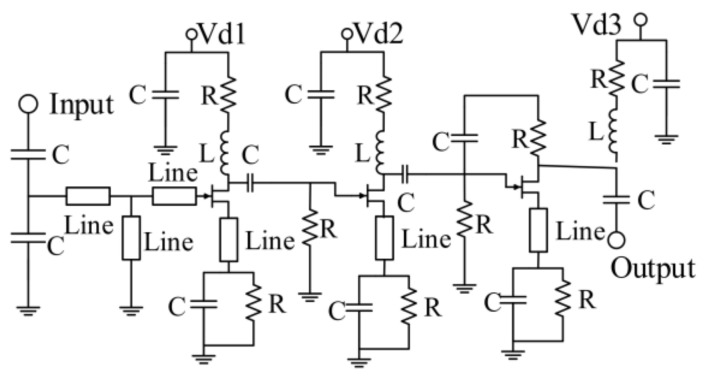
Simplified schematic diagram of the designed three-stage amplifier.

In terms of the amplifier design, our team has designed and processed a Ka-band amplifier chip based on the same process [49], so the structure of the amplifier in this section is basically the same as the one designed before. Due to the restrictions on the size of the chip, the amplifier is a three-stage amplifier, which is different from the four-stage amplifier processed before. Although the gain of the three-stage amplifier is reduced, when the six-port chip is used as a correlator, an additional amplifier is connected to its four-way output port. The function of the additional amplifier is to amplify the RF signal and provide reverse isolation between the detection circuit and the six-port chip, thereby preventing the influence of the mismatch of the detection circuit on the six-port chip. Therefore, the three-stage amplifier is sufficient for the designed six-port chip. The schematic diagram of the designed amplifier is represented in Figure 7.

Before the signal enters the chip, it passes through two low-noise amplifiers to meet the noise specification of the system. In addition, the system receives a noise signal; although it has been amplified by the amplifier, the power entering the chip is still low and cannot reach the $P_{1dB}$ of the amplifier. Therefore, the noise figure and $P_{1dB}$ of the designed amplifier is not the key point.

The main function of the amplifier in an integrated six-port chip is to amplify the input signal and compensate for the signal loss caused by the phase shifter and six-port network. The transistors of the designed amplifier with a size of 6 × 29 µm, 4 × 28 µm, and 2 × 60 µm are used for the first, second, and third stages, respectively. In addition, +3 V DC voltage is used for powering up all the stages, and 2.7 pF by-pass capacitors are also used.

**Figure 8:**
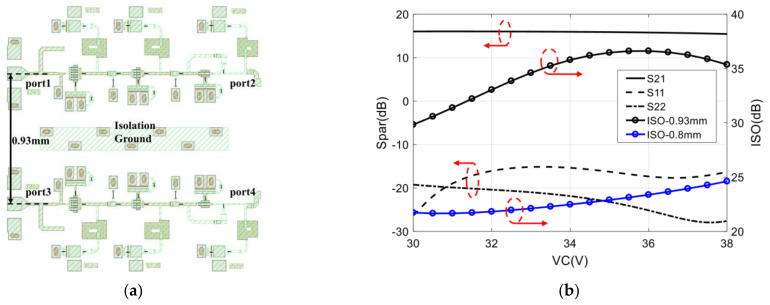
The layout design and the simulation results of the proposed two amplifiers: (**a**) The production stage layout design; (**b**) The simulation results.

It has been analyzed that the isolation between two amplifiers is important to the analog complex correlator, and the width of the designed chip is only 2 mm. In order to increase isolation between two input amplifiers, the distance between the input ports of the two amplifiers is as far as possible, and an isolation ground is also added in the middle of the two amplifiers. Therefore, the production stage layout design and the simulation results of the proposed two amplifiers are shown in Figure 8.

It can be seen that, from 32–36 GHz, the input return loss ($S_{11}$) is below −15 dB, the output return loss ($S_{22}$) is better than −20 dB, the gain ($S_{21}$) is larger than 15.7 dB, and the in-band gain fluctuation is less than 0.3 dB. In addition, when the distance between two input ports is 0.93 mm, the isolation between two amplifiers, which can be calculated through $dB\left(S_{21}\right)-dB\left(S_{41}\right)$, is larger than 30 dB. When the distance between two input ports is reduced to 0.8 mm, the isolation between the two amplifiers will deteriorate to 21 dB. Therefore, the distance between the two amplifiers should be kept at 0.93 mm to obtain greater isolation.

### 3.2. Design of the Phase Shifter

As discussed in Section 2.3, the PSR of the designed phase shifter needs to be greater than 180°. Moreover, to get a complete correlation circle, the phase shift of the designed phase shifter is required to be continuously variable; therefore, the analog phase shifter is selected to meet the requirements. In addition, the PSRE requirements of the proposed phase shifter are less than 19°; this special requirement is relatively rare in general phase shifters [50,51,52,53,54,55].

**Figure 9:**
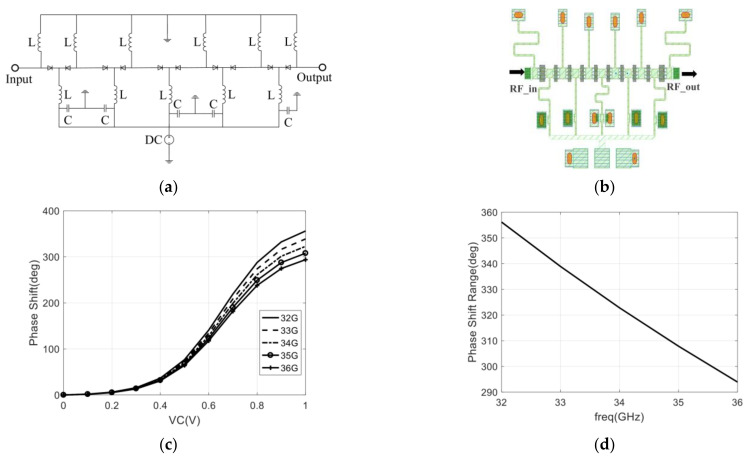
Phase shifter with only high-pass structure: (**a**) The schematic diagram of designed phase shifter; (**b**) The layout of designed phase shifter; (**c**) Simulation result of the phase-shifting with control voltage varying from 0 to 1 V; (**d**) Phase shift range with the control voltage is 1 V.

The designed phase shifter adopts a combined structure of high-pass phase-shifting and low-pass phase-shifting to minimize PSRE, which is different from the phase shifter that usually only uses a high-pass phase-shifting structure [51]. In order to evaluate the optimization of the designed phase shifter with high-pass and low-pass structures to PSRE, a phase shifter with a high-pass structure is designed first, which is shown in Figure 9.

It can be seen that the high-pass structure phase shifter utilizes 10 diodes. In the case of control voltage varying from 0 to 1 V, the maximum of the PSR is 356° @ 32 GHz, the minimum of the PSR is 294° @ 36 GHz, and the calculated PSRE is 62°. Moreover, in Figure 9d, we can see that the phase shift range of the designed phase shifter changes linearly with the frequency.

**Figure 10:**
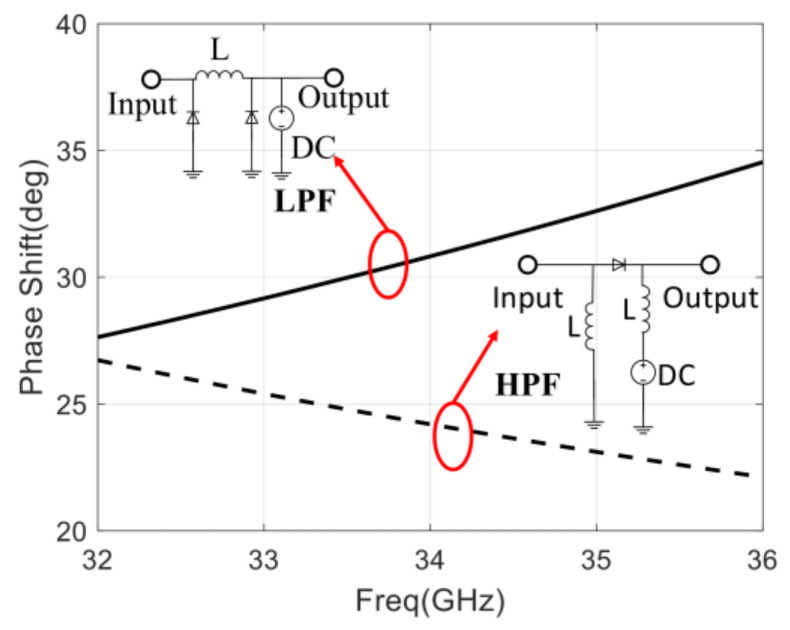
Phase shift range of high-pass and low-pass phase-shifting unit with control voltage varying from 0 to 1 V.

In order to optimize the PSRE, a phase shifter with high-pass and low-pass structures is proposed. As shown in Figure 10, the broadband phase-shifting range of the high-pass and low-pass phase-shifting units are compared, and the results show that the phase-shifting ranges of these two phase-shifting units are complementary in frequency. Therefore, a phase shifter with a high-pass and low-pass structure meets the requirements of the system for PSRE.

**Figure 11:**
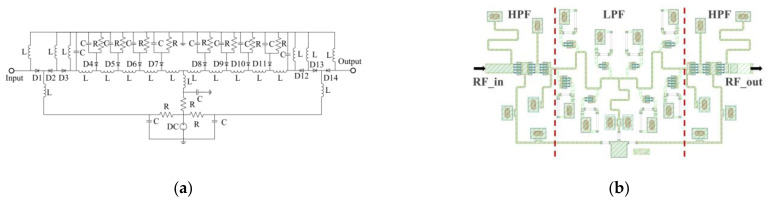
Phase shifter with high-pass and low-pass structure: (**a**) The schematic diagram of the designed phase shifter; (**b**) The layout of the designed phase shifter.

The simplified schematic diagram of the designed phase shifter with high-pass and low-pass structures is shown in Figure 11. From Figure 11a, the diodes of D4-D11 are low-pass phase-shifting structures, and other diodes are high-pass phase-shifting structures. Then, the production stage layout design of the proposed phase shifter is shown in Figure 11b. The size of D2 and D13 diodes are 4 × 20 µm, D5 and D10 diodes are 2 × 35 µm, D6 and D9 diodes are 2 × 25 µm, D7 and D8 diodes are 2 × 20 µm, and the rest of the diodes are 4 × 25 µm.

**Figure 12:**
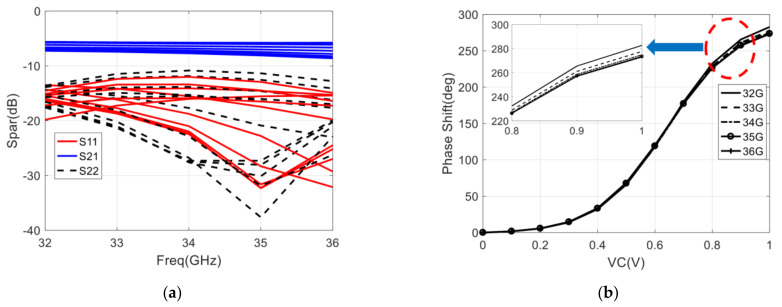
Simulation of the phase shifter: (**a**) The return loss and the insertion loss with the control voltage varying from 0 to 1 V; (**b**) Phase shift range.

The simulation result of the designed phase shifter is shown in Figure 12, and the operating frequency range of the phase shifter is 32–36 GHz. It is worth noting that the state of the phase shifter will change under different control voltages, so there will be different S-parameter simulation results under different control voltages. As shown in Figure 12a, with the control voltage varying from 0 to 1 V, the input and output return loss ($S_{11}$ and $S_{22}$) is better than −10 dB, and the insertion loss ($S_{21}$) varies from −5.5 to −7.5 dB. In addition, it can be seen that the maximum of the PSR is 283° @ 32GHz, the minimum of the PSR is 273° @ 36 GHz, which meets the 180° phase shift requirement, and the calculated PSRE is 10°, which is only 1/6 of the phase shifter with only low-pass structure.

### 3.3. Design of the Six-Port Network

**Figure 13:**
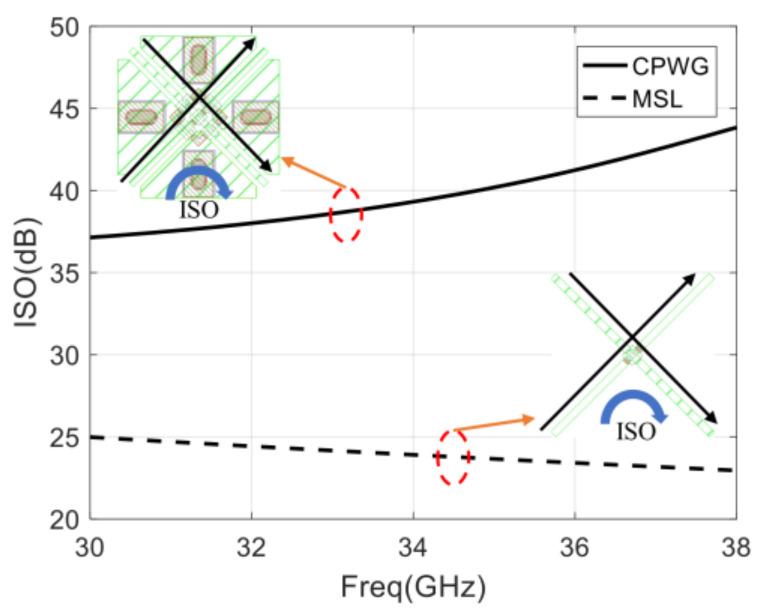
Isolation of the cross-over structure with CPWG and microstrip line.

The six-port network is a passive microwave system composed of couplers and power dividers. As shown in Figure 2, a cross-over structure is used in the six-port network to keep the four outputs on the same side. In order to increase the isolation of the cross-over structure, a CPWG transmission line is used instead of a microstrip line (MSL), which is represented in Figure 13.

It can be seen that the isolation of the CPWG transmission line is larger than 38 dB from 32–36 GHz and meets the requirements of the system. Compared with the microstrip transmission line, the isolation is improved by 13 dB.

**Figure 14:**
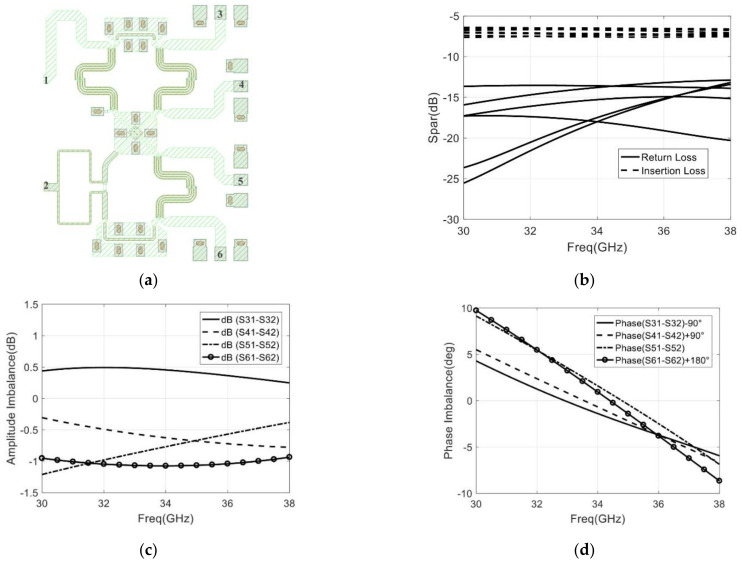
The designed six-port network: (**a**) Layout design; (**b**) Simulation of return loss and insertion loss; (**c**) Simulation of the amplitude imbalance; (**d**) Simulation of the phase imbalance.

In this section, the designed six-port network consists of a Wilkinson power divider and three Lange couplers. Therefore, the layout and simulation of the designed six-port is represented in Figure 14.

In the operating frequency of 32–36 GHz, the return loss of each port (($S_{11}$) to ($S_{66}$)) is below −13 dB, and the insertion loss (($S_{31}$ & $S_{32}$) to ($S_{61}\;$ & $\;S_{62}$)) varies from −6.5 to −7.5 dB, which is shown in Figure 14b. From Figure 14c,d, the amplitude imbalance is less than ±1 dB, and the phase imbalance is less than ±5.3°.

### 3.4. Simulation of the Integrated Six-Port Chip

**Figure 15:**
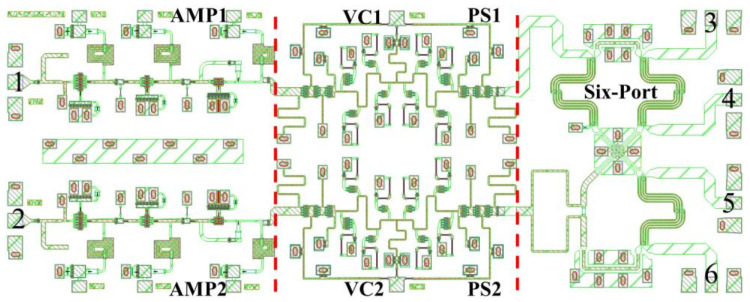
The layout design of the integrated six-port chip with a size of 5 mm × 2 mm. Numbers 1 to 6 represent the port number of the chip.

According to Figure 2, the layout of the integrated six-port chip can be obtained by combining the three circuits (Amplifier, Phase Shifter, and Six-Port) that have been designed above. The layout of the designed integrated six-port chip is shown in Figure 15, with a chip size of 5 mm × 2 mm.

**Figure 16:**
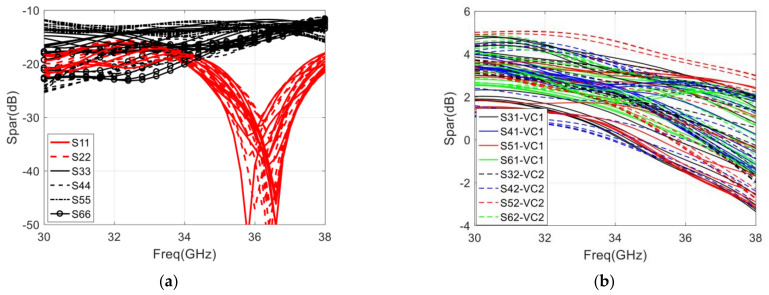

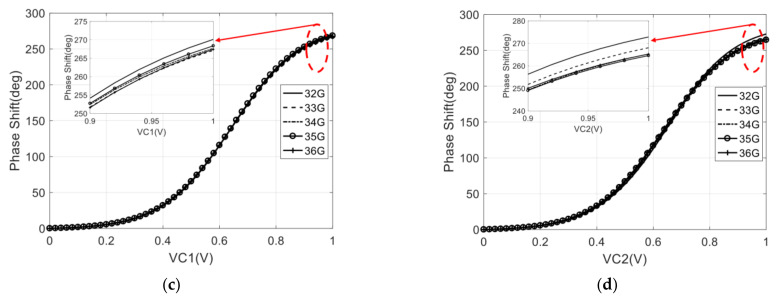
Simulation result of the integrated six-port chip with control voltage varying from 0 to 1 V: (**a**) Return loss of each port; (**b**) The gain of four output ports; (**c**) Phase shift range with control voltage (VC1) variances; (**d**) Phase shift range with control voltage (VC2) variances.

In order to obtain a complete correlation circle, it is necessary to change the voltage (VC1 and VC2, shown in Figure 15) of the phase shifter. As a result, the performance of the designed six-port chip under different control voltages should be given special attention. Therefore, the simulation result of the designed integrated six-port chip is shown in Figure 16.

In the 32–36 GHz frequency range, the return loss of input ports (($S_{11}$) and ($S_{22}$)) with the control voltage varying from 0 to 1 V is below −15 dB, as shown in Figure 16a. It should be noted that the return loss of port 1 varies with the control voltage VC1 and remains unchanged with the change of VC2. Therefore, only the return loss of port 1 with the change in VC1 is given in Figure 16a, and the return loss of port 2 is treated the same way. Furthermore, the return loss of output ports (($S_{33}$)) to ($S_{66}$)) with the control voltage varying from 0 to 1 V is below −13 dB. In Figure 16b, we can see that the gain of the four output ports varies from −2 to 5 dB with the control voltage varying from 0 to 1 V. The PSR of two-phase shifters, shown in Figure 16c,d, respectively, is larger than 265° with the control voltage varying from 0 to 1 V, and the calculated PSRE is 9°. In addition, the amplitude and phase imbalance of four output ports also varies with the control voltage (both VC1 and VC2) varying from 0 to 1 V. Due to the huge amount of data on this technical indicator, this article only gives a statistical result. The amplitude imbalance is less than ±5 dB, and the imbalance is less than ±15° with the control voltage (both VC1 and VC2) varying from 0 to 1 V.

**Figure 17:**
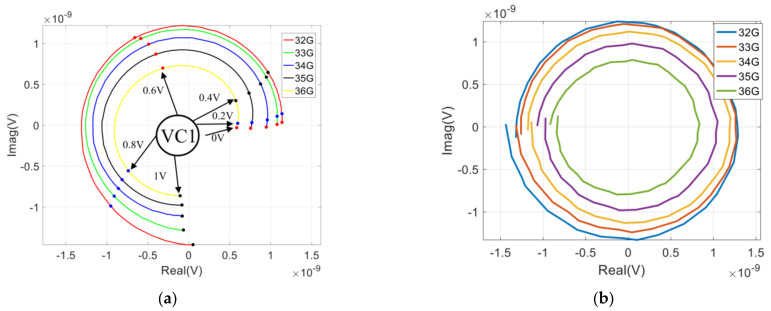
The simulation of the correlation circle: (**a**) The control voltage of only one phase shifter (VC1) varies; (**b**) The control voltage of both phase shifters varies.

Assuming the detector and differential amplifier behind the designed chip are ideal, we can obtain the correlation circle by changing the control voltage of the phase shifter. The simulation of the correlation circle is represented in Figure 17.

As represented in Figure 17a, the correlation circle is not closed by using only one control voltage (VC1) of the phase shifter, and the phase difference between two input signals varies from 0° to 265°. Moreover, a complete correlation circle can be formed by selecting both control voltages (VC1 and VC2), which is shown in Figure 17b, and the phase difference between the two input signals vary from −180° to 180°.

## 4. Measurement of the Integrated Six-Port Chip

**Figure 18:**
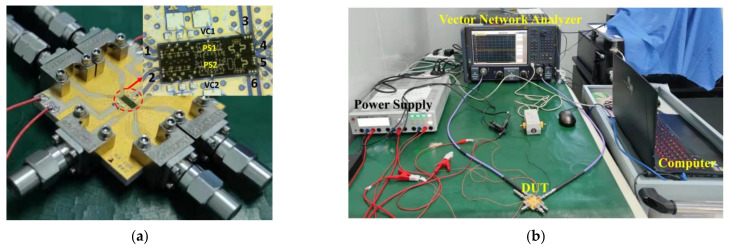
The photograph of the fabricated chip and test platform: (**a**) The microphotograph of the fabricated integrated six-port chip and test fixture; (**b**) The photograph of the automatic test platform for the integrated six-port chip.

The microphotograph of the fabricated integrated six-port chip is shown in Figure 18a, with a chip size of 5 mm × 2 mm, and a printed-circuit-board test fixture, which is designed using a 5-mil thickness Rogers 3003 substrate with 50 Ω microstrip, is taken for the measurement of the integrated six-port chip. Bond-wires are constructed for the interconnections of the chip and the input–output microstrip transmission lines. High-frequency K connectors and matching loads are employed for the measurements. As required by the chip, the DC voltage of the amplifier is 3 V, and the total consumed current is 73 mA.

The Keysight N5227B vector network analyzer and Rohde & Schwarz HMP 2030 programmable power supply are used for the measurement, shown in Figure 18b.

### 4.1. Measurement of the Return Loss, Gain, and Phase Shift Range

**Figure 19:**
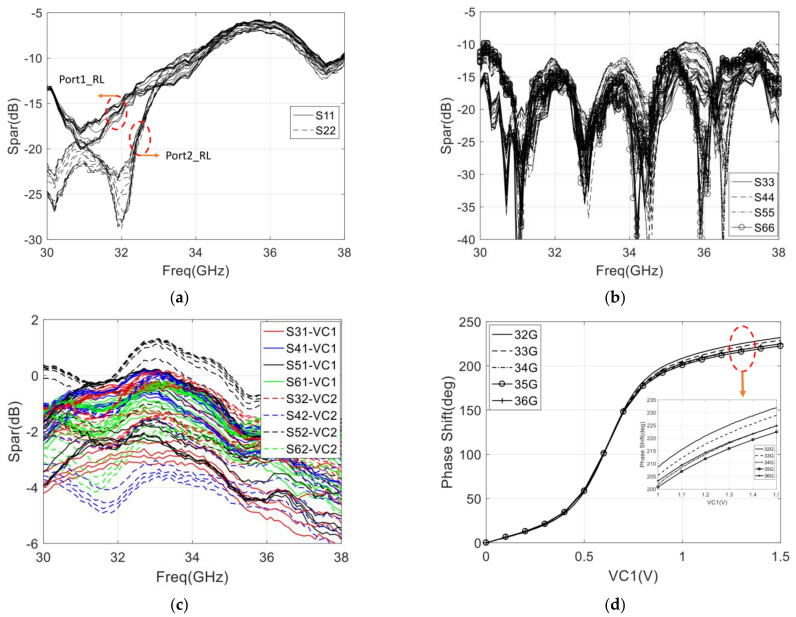
Simulation result of the integrated six-port chip with control voltage varying from 0 to 1 V: (**a**) Return loss of each port; (**b**) The gain of four output ports; (**c**) Phase shift range with control voltage (VC1) variances; (**d**) Phase shift range with control voltage (VC2) variances.

The measurement result of return loss, gain, and phase shift range is represented in Figure 19. To measure the phase-shifting curve of the integrated six-port chip, we can observe the phase of the output port signal by changing the control voltage of the phase shifter. Because the six-port chip has four output ports, four phase-shifting curves can be measured, and the final phase-shifting curve can be obtained by averaging these four curves.

The final phase-shifting curve of PS1 (which is described in Figure 18a) is shown in Figure 19d. It can be seen that the PSR is larger than 200° with the control voltage varying from 0 to 1 V, and the PSR can still be increased to 222° by the control voltage varying from 1 to 1.5 V, and the PSR of phase shifter meets the 180° phase shift requirement. Therefore, the calculated PSRE is 8.2° and 9.5° with the control voltage varying from 0 to 1 V and 0 to 1.5 V, respectively. The phase shift range of the phase shifter is still increasing when the control voltage is greater than 1 V and is basically unchanged when the control voltage is greater than 1.5 V. It is recommended that the control voltage range of the phase shifter is 0 to 1.5 V during practical application. The range of control voltage is also used in the measurement of return loss and gain.

Since the state of the designed chip is different under different control voltages, it is necessary to measure the S-parameters under each control voltage to evaluate the overall performance of the designed chip, and Figure 19a–c show the measured return loss and insertion loss under different control voltages. As shown in Figure 19a, the return loss of input ports with control voltage varying from 0 to 1.5 V is below −10 dB with the frequency range of 32–34 GHz, and the return loss is below −5.9 dB with the frequency range of 32–36 GHz. It can be seen that the return loss of the input port is deteriorated due to the frequency offset. Moreover, the return loss of output ports with control voltage varying from 0 to 1.5 V is below −10 dB, which is shown in Figure 19b. The return loss of four output ports is basically consistent with the simulation results. In Figure 19c, we can see that the gain of four output ports varies from −5 to 1 dB with the control voltage varying from 0 to 1.5 V. From the statistics of the measurement, the gain is about 4 dB lower than that of the simulation.

Compared with the simulation result, the measured return loss of the designed chip has deteriorated, especially in frequencies above 34 GHz. The reason for the return loss deterioration and gain reduction is that there is a deviation between the simulation model and actual parameters in the Ka-band when the chip is fabricated using 0.15-µm GaAs pHEMT technology.

In addition, the statistical result of the amplitude imbalance is less than ±4 dB, and the phase imbalance is less than ±20° with the control voltage (both VC1 and VC2) varying from 0 to 1.5 V, which is not shown in the figure of this section.

### 4.2. Measurement of the Isolation between Two Amplifiers

In order to measure the isolation between two amplifiers of the six-port chip, the influence of isolation between two amplifiers on four output ports of the six-port chip is analyzed first. Assuming that the inputs are two single-frequency signals $S_{1}\left(t\right)$ and $S_{2}\left(t\right)$, the voltage coupling coefficient between the two amplifiers is α, and the phase shift angles of PS1 and PS2 are $\varphi_{1}$ and $\varphi_{2}$, respectively. Therefore the two input signals of the six ports can be expressed as:

(16) $$\begin{array}{l} {S^{\prime }}_{1}=\sqrt{1-\alpha^{2}}e^{j\varphi_{1}}S_{1}+\alpha e^{j\varphi_{1}}S_{2}\\ {S^{\prime }}_{2}=\alpha e^{j\varphi_{2}}S_{1}+\sqrt{1-\alpha^{2}}e^{j\varphi_{2}}S_{2} \end{array}$$

If only the control voltage (VC2) of the PS2 is changed, and the signal is only provided at port 1, the value of $\varphi_{1}$ and $S_{2}\left(t\right)$ is 0. Then, the signals of the four output ports can be expressed as:

(17) $$\begin{array}{l} S_{31}=\sqrt{1-\alpha^{2}}S_{1}+\alpha e^{j\left(\varphi_{2}+90^{\circ }\right)}S_{1}\\ S_{41}=\sqrt{1-\alpha^{2}}e^{j\left(90^{\circ }\right)}S_{1}+\alpha e^{j\left(\varphi_{2}\right)}S_{1}\\ S_{51}=\sqrt{1-\alpha^{2}}e^{j\left(90^{\circ }\right)}S_{1}+\alpha e^{j\left(\varphi_{2}+90^{\circ }\right)}S_{1}\\ S_{61}=-\sqrt{1-\alpha^{2}}S_{1}+\alpha e^{j\left(\varphi_{2}\right)}S_{1} \end{array}$$

Similarly, if only the control voltage (VC1) of the PS1 is changed, and the signal is only provided at port 2, the value of $\varphi_{2}$ and $S_{1}\left(t\right)$ is 0, and the signals of the four output ports can be expressed as:

(18) $$\begin{array}{l} S_{32}=\sqrt{1-\alpha^{2}}e^{j\left(90^{\circ }\right)}S_{2}+\alpha e^{j\left(\varphi_{1}\right)}S_{2}\\ S_{41}=\sqrt{1-\alpha^{2}}S_{2}+\alpha e^{j\left(\varphi_{1}+90^{\circ }\right)}S_{2}\\ S_{51}=\sqrt{1-\alpha^{2}}e^{j\left(90^{\circ }\right)}S_{2}+\alpha e^{j\left(\varphi_{1}+90^{\circ }\right)}S_{2}\\ S_{61}=\sqrt{1-\alpha^{2}}S_{2}+\alpha e^{j\left(\varphi_{1}\right)}S_{2} \end{array}$$

It is noteworthy that the above derivation only considers the amplitude coefficient of the coupled signal and does not include the phase of the coupled signal. However, the isolation between the two amplifiers can still be estimated by calculating the maximum amplitude or phase error of the output port signal by changing the phase of the phase shifter, which can be expressed as:

(19) $$\begin{array}{l} A_{max}=20\log\left(\frac{\sqrt{1-\alpha^{2}}+\alpha }{\sqrt{1-\alpha^{2}}-\alpha }\right)\\ \Psi_{max}=2*\arctan\left(\frac{\alpha }{\sqrt{1-\alpha^{2}}}\right) \end{array}$$

where $A_{max}$ and $\Psi_{max}$ are the maximum amplitude error and phase error, respectively. As the value range of α is 0 to 1, we can replace *α* with sinδ, and the value range of δ is 0 to π/2, then Equation (19) can be simplified as:

(20) $$\begin{array}{l} A_{max}=20\log\left(\frac{\cos\delta +\sin\delta }{\cos\delta -\sin\delta }\right)\\ \Psi_{max}=2*\arctan\left(\tan\delta \right) \end{array}$$

From Equations (8), (19) and (20), the isolation between two amplifiers can be expressed as:

(21) $$\begin{array}{cc} ISO & =-20\log\left(\sin\left(\arctan\left(10^{\frac{A_{max}}{20}}\right)-45^{\circ }\right)\right)\\ & =-20\log\left(\sin\left(\frac{\Psi_{max}}{2}\right)\right) \end{array}$$

**Figure 20:**
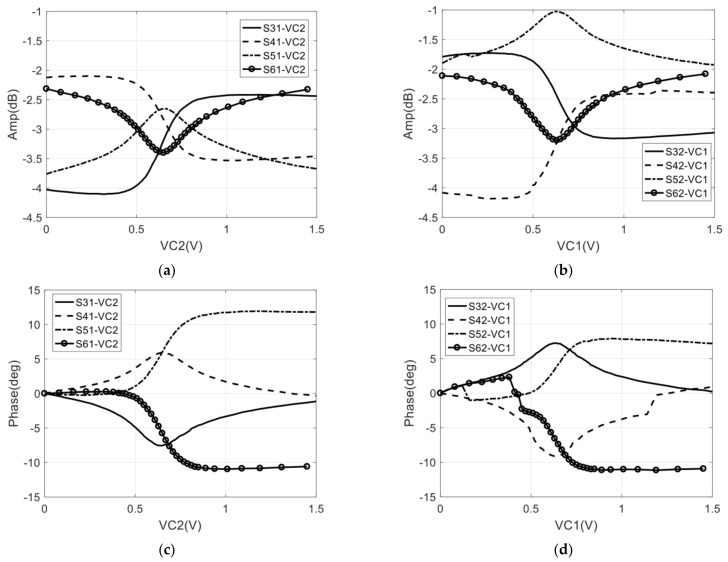
Measurement of the amplitude and phase fluctuation with the change in the control voltage @ 34 GHz: (**a**) Amplitude fluctuation with change of VC2 and input of port 1; (**b**) Amplitude fluctuation with change of VC1 and input of por t2; (**c**) Phase fluctuation with change of VC2 and input of port 1; (**d**) Phase fluctuation with change of VC1 and input of port 2.

In the measurement stage, the control voltage (VC2) of the PS2 is changed, as shown in Figure 18a, when the signal is provided at port 1 and not provided at port 2, and the isolation between the two amplifiers can be determined by measuring the change of the amplitude and phase of the signal at the output port. The isolation can be determined by changing VC1 and providing a signal to port 2 as well. The measurement of the amplitude and phase fluctuation with the change in the control voltage (VC1 or VC2) is shown in Figure 20.

As shown in Figure 20, in the case of change of VC2 and input of port 1 or change of VC1 and input of port 2, the amplitude and phase of the output signals of the four ports will change with the control voltage. It means that the isolation between the two amplifiers can be calculated through the maximum amplitude/phase error of four output signals.

**Figure 21:**
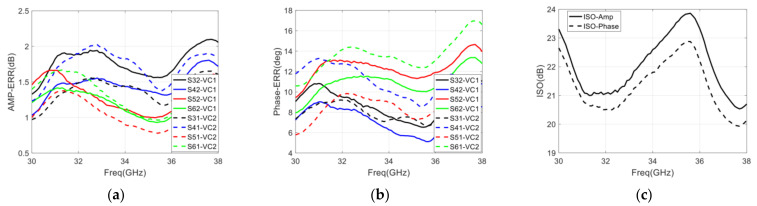
The maximum amplitude/phase error and calculated isolation between two amplifiers: (**a**) Maximum amplitude error of four output ports; (**b**) Maximum phase error of four output ports; (**c**) The calculated average isolation through maximum amplitude and phase error, respectively.

In the 32–36 GHz frequency range, the maximum amplitude/phase error of four output ports and the calculated isolation are shown in Figure 21. As shown in Figure 21c, the calculated average isolation is larger than 20 dB, and the isolation calculated by $A_{max}$ and $\Psi_{max}$ has good consistency.

Then, the comparison between simulation and measurement of the designed integrated six-port chip is shown in Table 1. It can be seen that, compared with the simulation results, the measurement results of various indicators have a certain degree of deterioration, but the most important indicators of PSR, PSRE, and the isolation between two amplifiers meet the requirements of the system.

Since this chip integrates functions such as phase-shifting and six-port circuits, it is compared with others’ work in terms of phase-shifting and six-port circuits, which is shown in Table 2 and Table 3.

## 5. Conclusions

In order to carry on the correlation processing directly to the RF signal and reduce the burden of LO link on the interferometric passive millimeter-wave imaging system, a Ka-band (32–36 GHz) six-port chip integrated with two amplifiers, two phase shifters, and a six-port network is designed and fabricated based on 0.15-µm GaAs pHEMT technology. At the design stage, the influence of the isolation between two amplifiers, PSRE, and isolation in the cross-over structure on the correlation results are analyzed and simulated. Then a wideband phase shifter with a low-pass and high-pass structure is designed, and the simulated PSR and PSRE of the phase shifter are 265° and 10°, respectively, with the control voltage varying from 0 to 1 V. Finally, the performance of the designed integrated six-port chip, with a size of 5 mm × 2 mm, is simulated. At the measurement stage, the return loss and gain of the chip have a certain degree of deterioration compared with the simulation result. With the control voltage varying from 0 to 1.5 V, the PSR and PSRE of the phase shifter are 222° and 10°, respectively, which meets the requirements of the system. The measurement method of isolation between two amplifiers is analyzed and verified, and the final measurement result of isolation is greater than 20 dB, which also meets the requirements of the system.

## Figures and Tables

**Table 1:** The comparison between simulation and measurement of the designed six-port chip.

Parameter	Simulation	Measurement
Control voltage (V)	0–1	0–1.5
Phase shift range (deg)	≥265	≥222
Phase shift range error (deg)	≤10	≤10
Isolation between two amplifiers (dB)	≥30	≥20

**Table 2:** Comparison table of the six-port circuit.

Reference	This Work	[29]	[32]
Process	0.15-µm GaAs	Waveguide	130 nm COMS
Freq (GHz)	32–36	15–20	60–65
Amplitude Imbalance (dB)	−2 ± 2.5 *	−6 ± 2.2	−6 ± 1
Phase Imbalance (deg)	±5 *	±15	±5
Return Loss (dB)	<−11 ^#^	<−12	<−12

* The control voltage of the six-port chip (VC1 and VC1) is 0 V. ^#^ The return loss of four output ports.

**Table 3:** Comparison table of phase shifters.

Reference	This Work	[52]	[54]	[55]
Process	0.15-µm GaAs	180-nm COMS	90-nm COMS	130-nm COMS
Freq (GHz)	32–36	15–20	57–64	21–24
PSR (deg)	≥222	360	360	≥290
PSRE (deg)	≤10	--	--	--
Gain (dB)	−2 ± 3	−5 ± 5	−8.5 ± 4.5	−7.4 ± 4.5

## Data Availability

Not applicable.
